# Microstructural Characterization of Al_0.5_CrFeNiTi High Entropy Alloy Produced by Powder Metallurgy Route

**DOI:** 10.3390/ma16217038

**Published:** 2023-11-04

**Authors:** Laura Elena Geambazu, Dorinel Tălpeanu, Robert Viorel Bololoi, Ciprian Alexandru Manea, Alina Elena Bololoi, Florin Miculescu, Delia Pătroi, Vasile Dănuţ Cojocaru

**Affiliations:** 1 Material Science and Engineering Faculty, National University of Science and Technology Politehnica Bucharest, Splaiul Independentei 313, 060042 Bucharest, Romania; laura.geambazu@icpe-ca.ro (L.E.G.); robert.bololoi@upb.ro (R.V.B.); ciprian.manea@icpe-ca.ro (C.A.M.); alina_elena.boarna@upb.ro (A.E.B.); florin.miculescu@gmail.com (F.M.); dan.cojocaru@upb.ro (V.D.C.); 2 National Institute for R&D in Electrical Engineering ICPE-CA Bucharest, Splaiul Unirii 313, 030138 Bucharest, Romania; delia.patroi@icpe-ca.ro

**Keywords:** mechanical alloying, high entropy alloys, powder metallurgy route, microstructural characterization

## Abstract

Alloys with superior properties represent the main topic of recent studies due to their effectiveness in reducing the cost of equipment maintenance and enhancing usage time, in addition to other benefits in domains such as geothermal, marine, and airspace. Al_0.5_CrFeNiTi was produced by solid state processing in a planetary ball mill, with the objective of obtaining a high alloying degree and a homogenous composition that could be further processed by pressing and sintering. The metallic powder was technologically characterized, indicating a particle size reduction following mechanical alloying processing when compared to the elemental raw powder materials. The microstructural analysis presented the evolution of the alloying degree during milling but also a compact structure with no major defects in the pressed and sintered bulk samples. The X-ray diffraction results confirmed the presence of face-centered cubic (FCC) and body-centered cubic (BCC) phases, predicted by the theoretical calculations, along with a hexagonal close-packed (HCP) phase, where the Al, Cr, Fe, Ni, and Ti phase was identified in both the alloyed powder material and sintered sample.

## 1. Introduction

High entropy alloy (HEA) materials have gained interest in recent years, being a main subject in multiple research studies due to their properties and effectiveness in aggressive environments. HEAs present specific core effects such as high entropy effect, cocktail effect, severe lattice-distortion effect, and sluggish diffusion effect which result, according to the literature [1,2], with superior properties such as high hardness and strength, high resistance to anneal softening, oxidation and wear resistance, high resistance to corrosion, and good magnetic and electric capabilities.

Due to their specific and unique effects, recent studies [3,4,5] present HEA as irradiation-resistant materials when comparing them with conventional alloys, leading to the possibility of utilizing them as structural materials for nuclear reactors [6].

Another advantage of the high entropy alloys is represented by the possibility of alloying the elements starting from the solid, liquid, or gas phase [7]. Alloys such as Al_x_CoCrFeNi [8], FeNiCrCuCo [9], and CrMnFeCoNi [10] obtained by the vacuum arc remelting method presented crystalline phases such as FCC and BCC, which, according to theory [11,12], results in properties such as ductility and high strength for the materials. When producing the high entropy alloys by the gas atomizing method, alloys such as Ni_6_Cr_4_WFe_9_Ti [13], AlCoCuFeNi [14], and Al_0.5_Cr_0.9_FeNi_2.5_V_0.2_ [15] present superior properties such as enhanced compressibility strength, ductility, and tensile strength.

Z.S. Nong et al. [16] reported that when AlCrFeNiTi HEA and AlCrFeNiTiMn_0.5_ HEA were synthesized by vacuum arc melting, dual BCC phases (AlNi_2_Ti-type B2 phase as BCC1 and (Cr, Fe)-based phase as BCC2) were identified by XRD analyses along with a Laves phase (Fe_2_Ti) for AlCrFeNiTi HEA. The calculated wear rate indicated improved wear resistance for the AlCrFeNiTi HEA when compared with the AlCrFeNiTiMn_0.5_ HEA.

Dual BCC phases (Ni, Al-type ordered BCC and disordered BCC) were reported by Liu et al. [17] when investigating Al0.5CrFeNiTi0.25 HEA produced by magnetic levitation melting using TEM (Transmission Electron Microscopy) techniques. The alloy presented tensile yield strength exceeding 755 MPa at 600 °C, indicating the potential of using a low-cost HEA in high-temperature applications.

Studies present the possibility of producing HEA by mechanical alloying and sintering into bulk samples in order to obtain electro-spark deposited coatings, with improved corrosion and wear resistance when compared with 316 L stainless steel [18,19].

Mechanical alloying was also reported as an elaboration method of CoCrFeNi HEA using a low ball-to-powder ratio (BPR) by A. Olejarz et al. [20]. It was observed that by increasing the milling time (10 h, 20 h, 30 h, and 40 h) at a constant 5:1 BPR, the number of contacts between the powder particles, balls, and vial increased, resulting in improved homogeneity of the material, more uniform distribution of the elemental materials, and grain size refinement.

In a study published by M. Reiberg et al. [21] regarding the AlCrFeNiTi HEA produced by mechanical alloying, the influence of reducing the aluminum content in the AlCrFeNiTi which resulted in a hardness increase was presented.

According to recent research [22], the alloying process of an equimolar AlCrFeNiTi high entropy alloy presented a similar microstructure to the analyzed samples mechanically alloyed for 20 h and 30 h. The alloy was consolidated by pressing at room temperature and sintering, presenting a structure with no major defects when microstructurally analyzed. Considering the results obtained, for this paper, the Al_0.5_CrFeNiTi high entropy alloy was mechanically alloyed for up to 20 h in order to obtain a desirable alloying degree, followed by the classic route of pressing and sintering, with the objective of obtaining bulk materials that can be further processed into different geometries. Samples from the alloyed material were collected at 5 h, 10 h, and 20 h in order to observe the alloying degree evolution.

This work was focused on developing materials with superior properties, as previously stated. The results obtained on AlCrFeNiTi equiatomic high entropy alloy metallic powder and bulk material developed in our previous research [22] led to the current paper. Al_0.5_CrFeNiTi was elaborated in order to compare the influence of the aluminum content in terms of alloying degree and bulk material structure under the same processing conditions. 

Due to factors such as erosion–corrosion, wear, high temperatures, and pressure present in aggressive media which could result in potential equipment failure, our research [18,19,22] was directed towards materials that are both economically efficient and with tailored properties, making high entropy alloys the ideal candidate. 

## 2. Materials and Methods

The Al_0.5_CrFeNiTi high entropy alloy was produced by the solid state processing method in order to obtain a high alloying and homogeneity degree in the entire mass. For the experiments, raw and pure (>99% purity) metallic powders of Al (~63 μm), Cr (~31.5 μm), Fe (~96.5 μm), Ni (~37 μm), and Ti (~200 μm) were homogenized and mechanically alloyed in a Planetary Ball Mill (Pulverisette 6, Frisch^®^ Classic line, Idar-Oberstein, Germany) for 20 h, with a ball to powder ratio (BPR) of 10:1 and a rotational speed of 300 RPM under an Ar atmosphere (Grade 5.0, Purity % >99.999). 

The process was performed in a stainless steel vial with stainless steel balls in order to avoid contamination of the mixture with other elements. The balls were of different dimensions (dia. 15 mm and dia. 20 mm) to increase the efficiency of the alloying process.

N-heptane was used as the process control agent, at a concentration of 2 wt%, reducing the unnecessary welding of the powder to the planetary ball mill vial and balls. Another benefit of wet milling is represented by reducing the milling time and increasing the alloying degree, which was observed during trials. 

**Figure 1:**
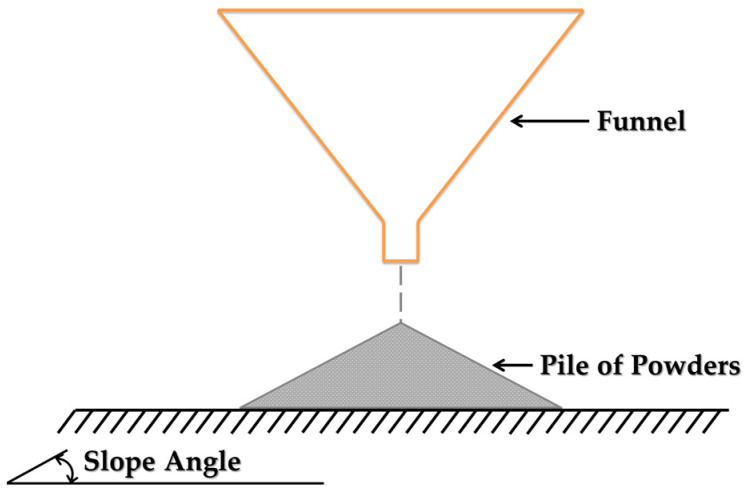
Schematic representation of slope angle and flow rate measurement setup.

After the mixture was alloyed, slope angle and flow rate were measured using a Carney funnel, represented schematically in Figure 1. The free flow density and tap density were measured in a glass graduated cylinder using 20 g of material. The metallic powder material was poured into the graduated cylinder and the volume was measured in order to determine the free flow density, followed by tapping the cylinder in order to determine the tap density of the content.

Particle size distribution was performed by sieving with a vibratory sieve shaker (Fritsch–Analysette 3^®^ SPARTAN, Idar-Oberstein, Germany), using 20 μm, 32 μm, 45 μm, 56 μm, 63 μm, 80 μm, 100 μm, 125 μm, 140 μm, and 160 μm aperture sieves. The average particle size was calculated using Equation (1), where *m* represents the percentage amount remaining on each sieve and *Dm* represents the average diameter between two consecutive sieves.

(1) $$\mathrm{Average}\;\mathrm{particle}\;\mathrm{size}=\frac{100}{\sum \frac{m}{\mathrm{Dm}}}$$

**Figure 2:**
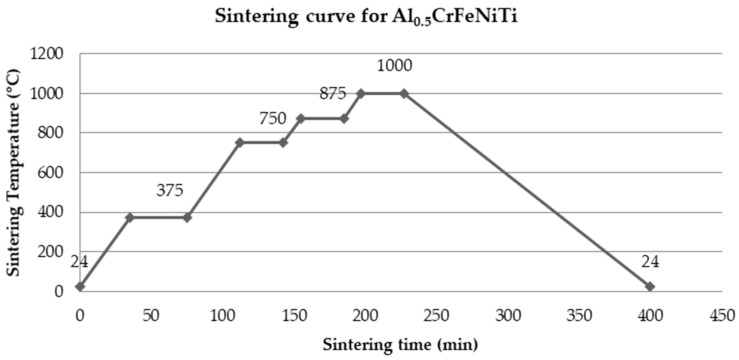
Sintering curve for Al_0.5_CrFeNiTi high entropy alloy.

The samples were further pressed by using Walter + Bai AG LFV-300 kN equipment with 200 kN pressing force with no bonding agent. Due to aluminum ductility, mold release wax was used as a coating on the interior of the die sleeve and contact surface of the pushing rods in order to avoid the adhesion of aluminum particles to the die set. The samples were pressed by using a stainless steel die set, resulting in compacts with 25 mm dia. and 6 mm thickness. The sintering process was performed in Multilab LAC/09 furnace equipment according to the sintering curve presented in Figure 2, with argon present in the furnace chamber as a protective gas. Starting from a room temperature of 24 °C, the temperature increased with a heating rate of 10 °C/min and a dwell time of 40 min at 375 °C for dewaxing of the samples. After each dwell time of 30 min at the temperatures 750 °C, 875 °C, and 1000 °C, a sample was removed from the furnace and cooled in air at room temperature. The last sample sintered at 1000 °C was cooled with the furnace. All 4 samples were structurally analyzed and the results were compared in order to establish the optimum sintering time for this research. 

Figure 2 presents the sintering curve for the Al_0.5_CrFeNiTi high entropy alloy.

Specimens were metallographically prepared by polishing with SiC paper followed by cleaning with high purity alcohol (ethyl alcohol 99.9%) and drying with hot airflow for microstructural and chemical analyses in order to observe the structure obtained by the sintering process.

The metallic powders and sintered specimens were analyzed by FEI Philips Scanning Electron Microscopy (SEM) with an EDAX Sapphire electron dispersive energy microscope (Portland, OR, USA).

X-ray diffraction analyses were performed by using the D8-Discover diffractometer (Bruker, Bremen, Germany), with Cu primary radiation (λ = 1.540598Å), 1D LynxEye detector on the secondary side, and Göebel mirror. The diffractograms were obtained with an angular increment of 0.04°, at a scanning speed of 1 s/step, and crystallographic phases were carried out by using the ICDD PDF 2 Release 2022 database and the structures were found to be similar to the indexed files PDF 01-071-4653 for nickel, PDF 01-089-2959 for titanium, PDF 03-065-4607 for chromium iron, PDF 01-081-9823 for aluminium, PDF 04-017-1425 for Chromium Iron Nickel Titanium Aluminium, PDF 04-001-3364 for Aluminum Titanium, PDF 01-083-3987 for Aluminum Nickel, and PDF 04-003-5565 for Chromium Titanium.

## 3. Results and Discussion

For designing the high entropy alloy studied in this work, theoretical calculations of enthalpy of mixing (Δ*H_mix_*), entropy of mixing (Δ*S_mix_*), atomic size difference (δ), solid solution formation parameter (Ω), and valence electron concentration (VEC) were performed using the following equations:

(2) $$\Delta H_{mix}=\sum_{i=1,i\neq j}^{N}\Omega_{ij\;}c_{i}c_{j}$$

(3) $$\Delta S_{\mathrm{mix}}=-R\sum_{i=1}^{n}{c\;}_{i}\cdot \ln c_{i}$$

(4) $$\delta =100\sqrt{\sum_{i=1}^{n}c_{i}{\left(1-\frac{r_{i}}{ṝ}\right)}^{2}}$$

(5) $$\Omega =\frac{T_{m}\cdot {\Delta S}_{mix}}{{|\Delta H}_{mix}|}$$

(6) $$\mathrm{VEC}=\sum_{i=1}^{n}{VEC\;}_{i}\cdot c_{i},$$

where $\Omega_{ij\;}=4\Delta$_AB_^mix^, Δ_AB_^mix^ is the binary liquid mixing enthalpy of AB alloys, $c_{i}$ and $c_{j}$ are the atomic fraction of specific components, *n* is the number of components, T_m_ is the melting point of the HEA, r is the atomic radius, ṝ is the average radius of the alloy calculated by $ṝ=\Sigma c_{i}r_{i}$, and R is the ideal gas constant.

The collective behavior of the HEA constituent elements can be used in order to attempt the prediction of crystal structures and solid solution phase over intermetallic compounds formation [23,24], where $\Delta H_{mix}$ calculated with Equation (2) is in the suitable range of −22 ≤ $\Delta H_{mix}$ ≤ 7 kJ/mol, Δ*S*_mix_ (Equation (3)) is in the suitable range of 11 ≤ Δ*S*_mix_ ≤ 19.5 J/(K·mol), and δ (Equation (4)) is in the suitable range of 0 ≤ δ ≤ 8.5. 

According to phase stability formation criteria calculated by X. Yang et al. [25], the solid solutions formation ability of the alloy could be estimated if Ω ≥ 1.1 when calculated with Equation (5).

Equation (6) was used for calculating the VEC, which, according to theory [24,26], if the calculated value is greater than 8, then an abundance of FCC solid solution structure is formed in the alloy, resulting in an improved ductility. When the VEC value is lower than 6.8, a majority of the BCC solid solution phase is formed, resulting in an improvement of the alloy’s strength. For a value between 6.8 and 8, a mixture of both phases will be present.

The calculated thermodynamic parameters for the Al_0.5_CrFeNiTi high entropy alloy are presented in Table 1. According to the obtained values, solid solutions are prone to form for the studied alloy.

Dominguez et al. [27] presented the possibility of complex phases at intermediate values to be identified for 6 < VEC < 8, due to the phase discrimination in a 2-dimensional plan. It was also mentioned that for 3 < VEC < 6, a single BCC phase was identified, and for 8 < VEC < 11, a single FCC phase was identified [28].

The VEC calculated value of 6.55 indicates the favoring of the predominant formation of the BCC phase but with the possibility of the presence of complex phases. 

**Figure 3:**
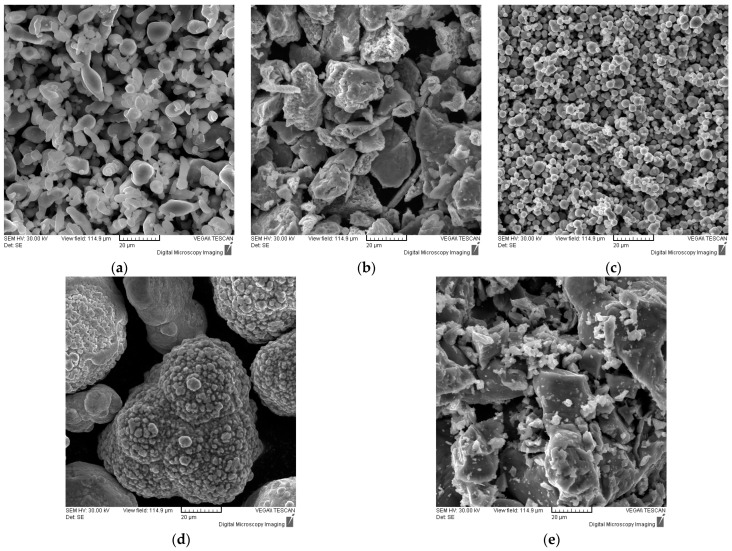
SEM analyses results for the raw elemental powders before mixing; (**a**) Al, (**b**) Cr, (**c**) Fe, (**d**) Ni, and (**e**) Ti.

After the theoretical calculations were performed, the raw metallic powders were SEM analyzed and the morphology of the individual materials is presented in Figure 3, where Figure 3a presents the morphology of Al, Figure 3b of Cr, Figure 3c of Fe, Figure 3d of Ni, and Figure 3e of Ti.

The morphologies of the raw powders present the different shapes and sizes of the selected components, where Fe and Ni present a more spherical shape, Cr and Ti present polygonal shapes, and Al presents particles of both spherical and polygonal shapes.

**Figure 4:**
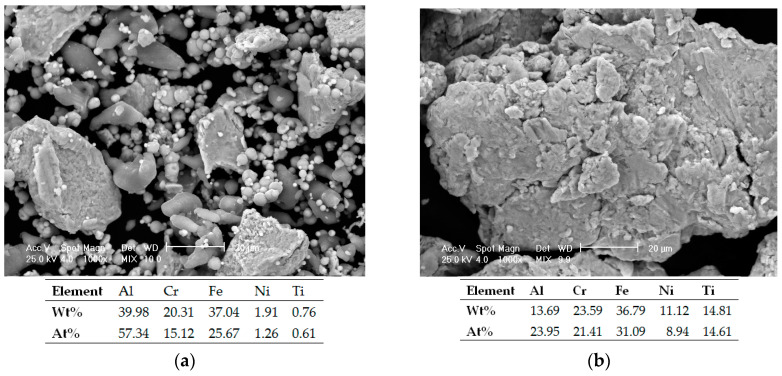

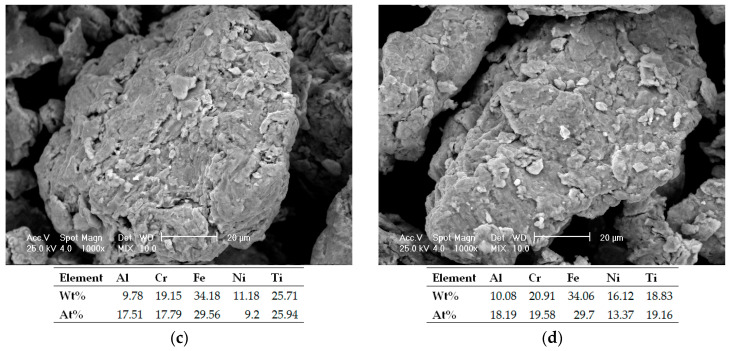
Al_0.5_CrFeNiTi high entropy alloy SEM and EDS analysis results (inset): mixed powders sample (**a**) and mixture alloyed for 5 h (**b**), 10 h (**c**), 20 h (**d**).

In order to obtain the high entropy alloy, the mixture of elemental materials was mechanically alloyed and samples were collected at different periods of time in order to observe the alloying degree evolution. In Figure 4a, the mixed powders sample was analyzed and the results are presented. The sample was composed of different shaped and sized particles specific for each raw metallic powder and the EDS analyses results confirm the mixture’s chemical composition.

The evolution of the alloying degree was studied and the microstructural and EDS analyses results are presented in Figure 4b,c. In Figure 4b, the incipient phase of the alloying process of the sample collected after 5 h of alloying in the planetary ball mill, where particles were welded and the size of the particles was changed, could be observed. The composition of the mixture was confirmed by the EDS analyses. Figure 4c shows an enhanced uniformity of the particle shape and size with a reduced Cr concentration in the analyzed area after 10 h of mechanical alloying. In Figure 4d, the microstructural analyses results of the alloyed samples present the mixture after 20 h of alloying. The particles were more uniform, reduced in size, and agglomerated when compared with the results from the beginning of the process, due to the high effectiveness of the wet milling and of the used equipment. When comparing the results with the research on AlCrFeNiTi HEA [22], a similarity when discussing the period of time required for the particles to agglomerate during the alloying process could be observed. The EDS analyses confirmed the alloy composition and although the O and C were not identified due to the detection limit of the used equipment, their presence is not excluded.

The mechanically alloyed Al_0.5_CrFeNiTi high entropy alloy was technologically characterized before further processing in order to establish the parameters for pressing and sintering. The results are presented in Table 2.

The first analysis consisted of the comparison of the flow rate between the elemental powders and the alloyed powder. The flow rate could be influenced by the shape of the particle, resulting in a better flow rate for the spherical-shaped Ni metallic powder compared with Al, Cr, and Fe.

For the mechanically alloyed powder, a flow rate value of 7.62 g/s was obtained, indicating an improvement when compared with the values of Al, Cr, Fe, and Ti powders but also when comparing the results with the 6.91 g/s flow rate reported for equiatomic AlCrFeNiTi HEA [22]. Free flow density and tap density analyses were performed as a comparison between the elemental metallic powders and the powder alloy in order to calculate the packing density. 

**Figure 5:**
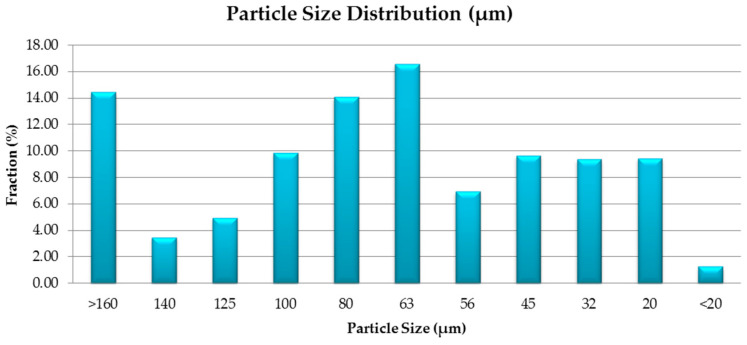
Particle size distribution for the Al_0.5_CrFeNiTi HEA obtained by mechanical alloying.

The results of the particle size distribution are presented in Figure 5.

The particle size distribution indicated that the highest fraction obtained was in the 56–80 µm range. The particle size was influenced by the presence of the Al and Ti elemental materials as ductile materials, but also by the micro-welding and fracturing of the particles during the mechanical alloying process. 

For Al_0.5_CrFeNiTi HEA, the calculated average particle size was 60.4 µm, being significantly reduced due to fracturing during mechanical alloying when compared with the elemental metallic Ti powders, with an average particle size of 200 µm, and Al, with an average particle size of 63 µm according to the manufacturer. 

**Figure 6:**
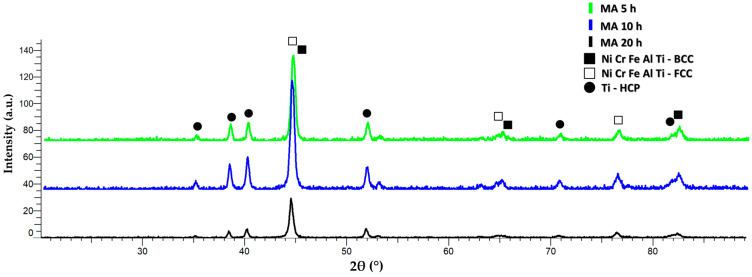
X-ray diffraction results for the Al_0.5_CrFeNiTi HEA mechanically milled sample for 5 h, 10 h, and 20 h.

Figure 6 presents the XRD analyses results for the samples of the Al_0.5_CrFeNiTi mixture milled for 5 h, 10 h, and 20 h in a planetary ball mill.

For the highest intensity peak, FCC and BCC phases were observed. At the 2θ angular position of 44.6°, a peak with all constituent elements was identified, where for the BCC phase, an abundance of CrFe was observed. The HCP phase with a titanium-like crystal structure was identified at 35.11°, 38.45°, 40.25°, 53.11°, 70.79°, and 82.41° at the 2θ angular position. It was observed that after 20 h of milling, the peaks’ intensity decreased and that they were shifted to a higher angular position compared to those at 5 h and 10 h of milling. This indicates a more refined structure and, in terms of the interstitial positioning of the elements, the XRD analysis suggests smaller lattice parameters with the development of the complex phases.

Based on the obtained results, the alloying degree was decided to be sufficient for the current case, and the metallic powder was pressed into green samples. Due to the ductility of the aluminum and titanium content, the samples were consolidated without the need for a bonding agent. After testing different pressing parameters, it was observed that by using a force of 200 kN, the samples could be handled, indicating good structural stability. The next step of the process was sintering the pressed sample in order to obtain bulk material for further processing. 

The first dwell at 375 °C for 40 min was performed due to residual organic matter resulting from the mold release wax used during the pressing process. 

**Figure 7:**
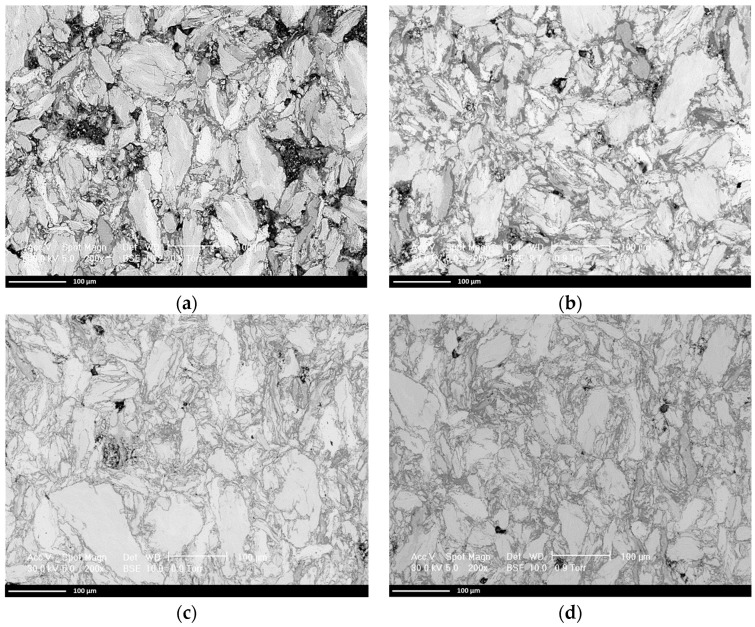
Microstructural analyses results for the sintered samples at (**a**) 750 °C cooled in air, (**b**) 875 °C cooled in air, (**c**) 1000 °C cooled in air, and (**d**) 1000 °C cooled with the furnace.

In Figure 7a, the analysis results for the Al_0.5_CrFeNiTi sintered at 750 °C, with 30 min dwell and cooling in air are presented. From the SEM microscopic analyses of the surface, the incipient stage of sintering bridges could be observed, but voids between particles of approx. 100 µm and pores were also present, resulting in a possible low mechanical strength compacted sample. The sintering behavior was tracked by microscopic analyses for samples sintered at 875 °C and cooled in air (Figure 7b), 1000 °C and cooled in air (Figure 7c), and 1000 °C and cooled with the furnace (Figure 7d). A gradual reduction of the voids and pore size was observed, where for the sample presented in Figure 7d, a better consolidation was present. 

The process was performed under an argon atmosphere and it was followed by polishing and cleaning of the samples. The samples were microstructurally analyzed and the results are presented in Figure 7.

Theoretical density, apparent density, and porosity were calculated in order to verify how the sintering temperature and the cooling process affected the compacted samples. The results of the calculations are presented in Table 3.

**Figure 8:**
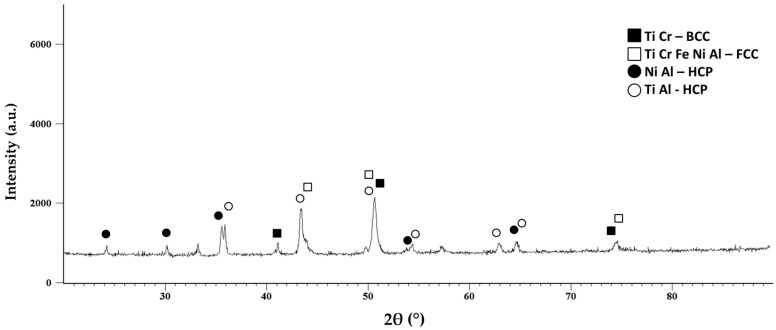
X-ray diffraction results for the Al_0.5_CrFeNiTi high entropy alloy sintered sample.

From the experimental data presented in Table 3, it resulted that the lowest porosity of 21.88% and the highest apparent density of 4.86 g/cm^3^ were obtained for Sample 2 (sintered at 875 °C and cooled in air). The SEM microstructural analyses presented a more refined microstructure for Sample 4 (sintered at 1000 °C and cooled with the furnace) with lower pore diameters, indicating an improved structure when compared with Sample 2. It was also observed in the SEM analyses results (Figure 7) that no cracks were present in the structure of the sintered samples. In Figure 8, the X-ray diffraction (XRD) analysis for Sample 4 is presented.

From the XRD analysis results, it was observed that the principal phases were found at the 2θ angular positions of 36°, 43.5°, and 51°, which are rich in NiAl and TiAl. For the peaks with the highest intensity, HCP, BCC, and FCC phases are present. At the 2θ angular position of 43.5°, a peak with all constituent elements was observed. BCC phases were observed at the 2θ angular positions of 41°, 51°, and 74.5°, confirming the theory with Cr Ti-like crystal structures. The identified FCC phases were to be expected due to the titanium and aluminum content. 

The VEC parameter has a decisive role when discussing the possible solid solution formation in the high entropy alloys according to Guo et al. [26]. For the present case, when comparing the VEC theoretical calculation results, the obtained value indicated an expected BCC phase, but when reducing the aluminum content, the FCC phase is favored to form, which is confirmed by the XRD analyses results for both the mechanically alloyed metallic powder and the sintered sample. The HCP phases are specific to the Ti α phase, which is mainly stabilized by the presence of aluminum and oxygen content [26]. This phenomenon could be determined by the high entropy alloy sintering temperature (1000 °C) being above the phase transformation temperature of titanium (882 °C) from α phase to β phase, where BCC phases are to be expected.

**Figure 9:**
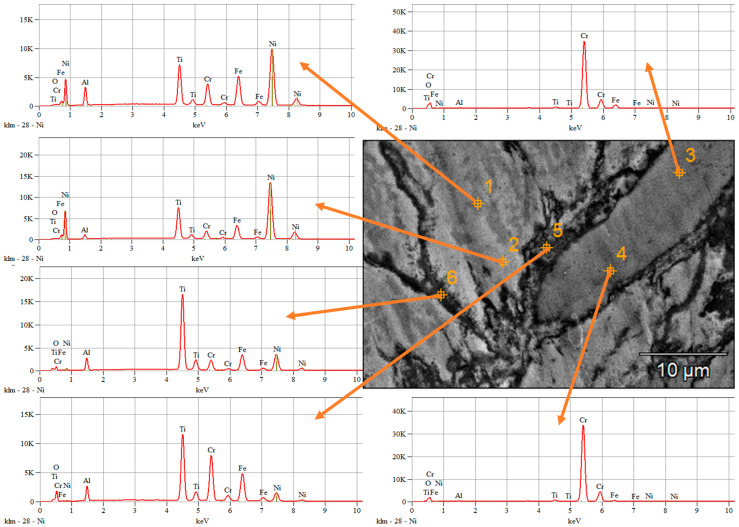
EDS analyses results at different points of the selected surface area.

The homogeneity degree of the sintered alloy (Sample 4) was analyzed at different points selected on the surface area of the sample, the arrows indicating the obtained spectra in each point and the results are presented in Figure 9.

For points 3 and 4, an abundance of Cr was observed, correlated with the data collected and presented in Table 4. Points 5 and 6 present an abundance of Ti, but as for points 3 and 4, the presence of all the component elements can be observed, indicating that agglomeration took place during the mechanical alloying process. Overall, when microscopically analyzing a small area of the bulk sample, the high entropy alloy is confirmed. For points 1 and 2 results analysis, areas with increased compositional homogeneity were observed, compared to points 3–6, where particular constituent elements are in abundance.

The chemical composition analyses indicated the presence of the initial mixture elements with no contamination. No traces of the used mold release wax were identified in the bulk materials.

The compositional analyses of each point (Table 4) indicate the oxidation of the sample, which occurred during the material processing and confirmed the chemical composition of the initial mixture of Al, Cr, Fe, Ni, and Ti. The oxidation was minimized due to the inert gas (argon atmosphere) used for the mechanical alloying process and sintering process, but also due to powder manipulation in a glovebox with a controlled atmosphere. From previous research [18], when using CoCrFeNiMo_0.5_ high entropy alloys as coatings deposited by electro spark deposition technique, there is a passivation tendency observed due to chromium segregation in the coating. Passive films are also to be expected due to the presence of Ni, according to M. Duarte et al. [29], resulting in an enhancement of the material corrosion resistance. 

According to the literature, when a PCA (Process Control Agent) is used for wet milling, C contamination is observed in the case of methanol, toluene [30], and Dodecane (0.7 wt%) [31]. H. Kotan et. al. [31] reported that the Cr_7_C_3_ phase was detected by TEM analyses when Dodecane (0.7 wt%) was used as PCA for the mechanical alloying, followed by spark plasma sintering (SPS) of the CoCrFeNi HEA doped with Zr/Y in a graphite die. The authors observed that the C contamination was present after the mechanical alloying and annealing process, but also that the HEA powder reacted with the graphite die set used for consolidating the samples.

R. Jayasree et al. [30] reported that for Al_x_CoCrFeNi HEA milled for 15 h with methanol and toluene as PCA, the XRD analyses indicated the presence of C traces, but due to the detection limit of the equipment, the C content could not be confirmed. Upon mechanically activated annealing, the Cr7C3 phase was identified and reported.

The PCA used for this work (2 wt%) contains approximately 84 wt% C and although it was not identified in the chemical composition after alloying, its presence is not excluded when correlating the results to the literature, due to the possibility of being under the limit of detection for the used equipment.

## 4. Conclusions

For this paper, the possibility of producing an Al_0.5_CrFeNiTi high entropy alloy produced by mechanical alloying and compacting by cold pressing and furnace sintering was explored. 

After analyzing the SEM and EDS analyses results for the Al_0.5_CrFeNiTi high entropy alloy produced by solid state processing, the effectiveness of the mechanical milling process was observed in terms of agglomeration of particles, particle shape, and size uniformity, with no contaminations detected. The technological characterization results indicated the effect of welding and fracturing of the elemental materials, where the calculated average particle size for the studied alloy was 60.4 µm. XRD analyses results indicate that BCC and FCC phases were identified along with the HCP phase, where for the peak with the highest intensity at the 2θ angular position of 44.6°, all the constitutive elemental components were present, highlighting that the alloying occurred.

The sintered samples’ microstructural analyses indicated that sintering the pressed sample at 1000 °C and cooling it with the furnace resulted in a compact material with no cracks and a porosity of 22.65%. Although the sample sintered at 875 °C and cooled in air resulted in 21.88% porosity, the pore size was larger. 

The XRD analyses for the sintered samples indicated the presence of FCC and BCC phases with the elemental component being identified at the 2θ angular position of 43.5°, along with HCP Ni Al and Ti Al-like crystal structures. As for the alloyed powder, the analyses confirm the VEC calculations. The chemical analyses results confirmed the mixture composition, but it was also observed that a slight oxidation occurred.

The results obtained in this manuscript represent the starting point of further processing methods in order to obtain a lower porosity degree for the bulk material.

## Figures and Tables

**Table 1:** The results of thermodynamic calculations for Al_0.5_CrFeNiTi HEA.

Parameter	Units	Value
$\Delta H_{mix}$	kJ/mol	−20.968
Δ*S*_mix_	J/(K·mol)	13.19
δ	-	7.451
Ω	-	1.122
VEC	-	6.55

**Table 2:** Technological analyses results for the elemental metallic powders and Al_0.5_CrFeNiTi high entropy alloy powder.

Material	Free Flow Density (g/cm^3^)	Tap Density (g/cm^3^)	Packing Density (%)	Free Flow (g/s)	Slope Angle (°)
Al	1.21	1.47	82.3	1.09	27.02
Cr	2.45	3.2	76.6	1.2	31.79
Fe	3.12	4.05	77.0	0.85	9.64
Ni	4.54	5.1	89.0	12.19	17.74
Ti	1.56	1.85	84.3	4.95	26.1
Al_0.5_CrFeNiTi	2.72	3.21	84.8	7.62	23.6

**Table 3:** Theoretical density, apparent density, and porosity data obtained for the sintered samples.

Sample	Sintering Temperature (°C)	Cooling	Theoretical Density (g/cm^3^)	Apparent Density ± Std. Dev. (g/cm^3^)	Porosity ± Std. Dev. (%)
Sample 1	750	in air	6.23	4.65 ± 0.004	25.34 ± 0.069
Sample 2	875	in air	6.23	4.86 ± 0.009	21.88 ± 0.142
Sample 3	1000	in air	6.23	4.76 ± 0.004	23.55 ± 0.075
Sample 4	1000	with the furnace	6.23	4.81 ± 0.010	22.65 ± 0.159

**Table 4:** EDS analyses results highlighting the elemental concentration on different surface points.

Location	Elemental Concentration											
	O		Al		Ti		Cr		Fe		Ni	
	wt%	at %	wt%	at %	wt%	at %	wt%	at %	wt%	at %	wt%	at %
Point 1	1.73	5.38	6.78	12.5	14.61	15.18	9.65	9.23	17.16	15.28	50.07	42.43
Point 2	1.08	3.6	2.2	4.35	15.39	17.14	4.75	4.87	10.09	9.63	66.49	60.41
Point 3	2.16	6.7	0.57	1.05	0.66	0.68	88.24	84.19	7.12	6.32	1.25	1.06
Point 4	2.19	6.77	0.54	0.99	0.75	0.77	93.28	88.65	2.14	1.89	1.1	0.93
Point 5	7.68	20.47	5.31	8.39	28.12	25.04	26.76	21.93	22.9	17.48	9.23	6.69
Point 6	5.33	14.75	5.87	9.62	42.88	39.63	7.96	6.77	15.82	12.53	22.14	16.7

## Data Availability

Not applicable.
